# Supplementary material for: Anticancer Activity of Plant Tocotrienols, Fucoxanthin, Fucoidan, and Polyphenols in Dietary Supplements
Source: Nutrients. 2024 Dec 11;16(24):4274. doi: 10.3390/nu16244274 (PMC11678266; doi:10.3390/nu16244274)
Supplement: Supplementary file 1 [file nutrients-16-04274-s001.zip › nutrients-3275168-supplementary.pdf]

Supplementary Material

4.0

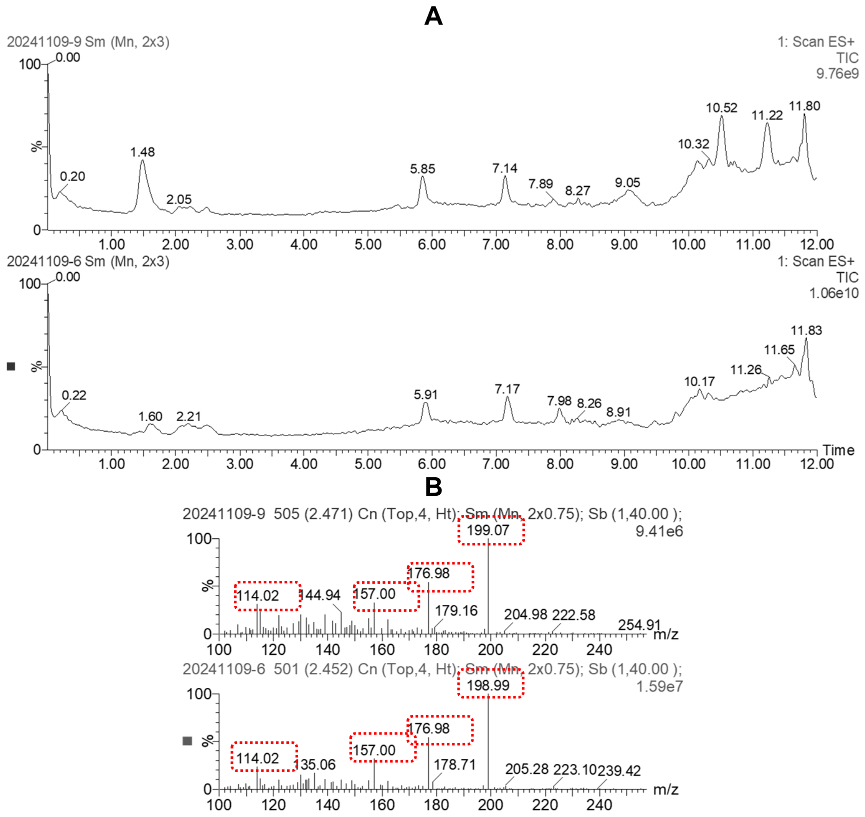

Figure S1. (A) LC-MS analysis (TIC-ESI+) of dietary supplement 4.0 (top) and the ThinOgen ingredient (bottom). **(B)** Extracted MS spectrum of selected peak at tR 2.4 min of each sample (the MS spectrum of supplement 4.0 is shown at the top, and ThinOgen at the bottom). Red boxes highlight the same ions present in both samples.

10.0

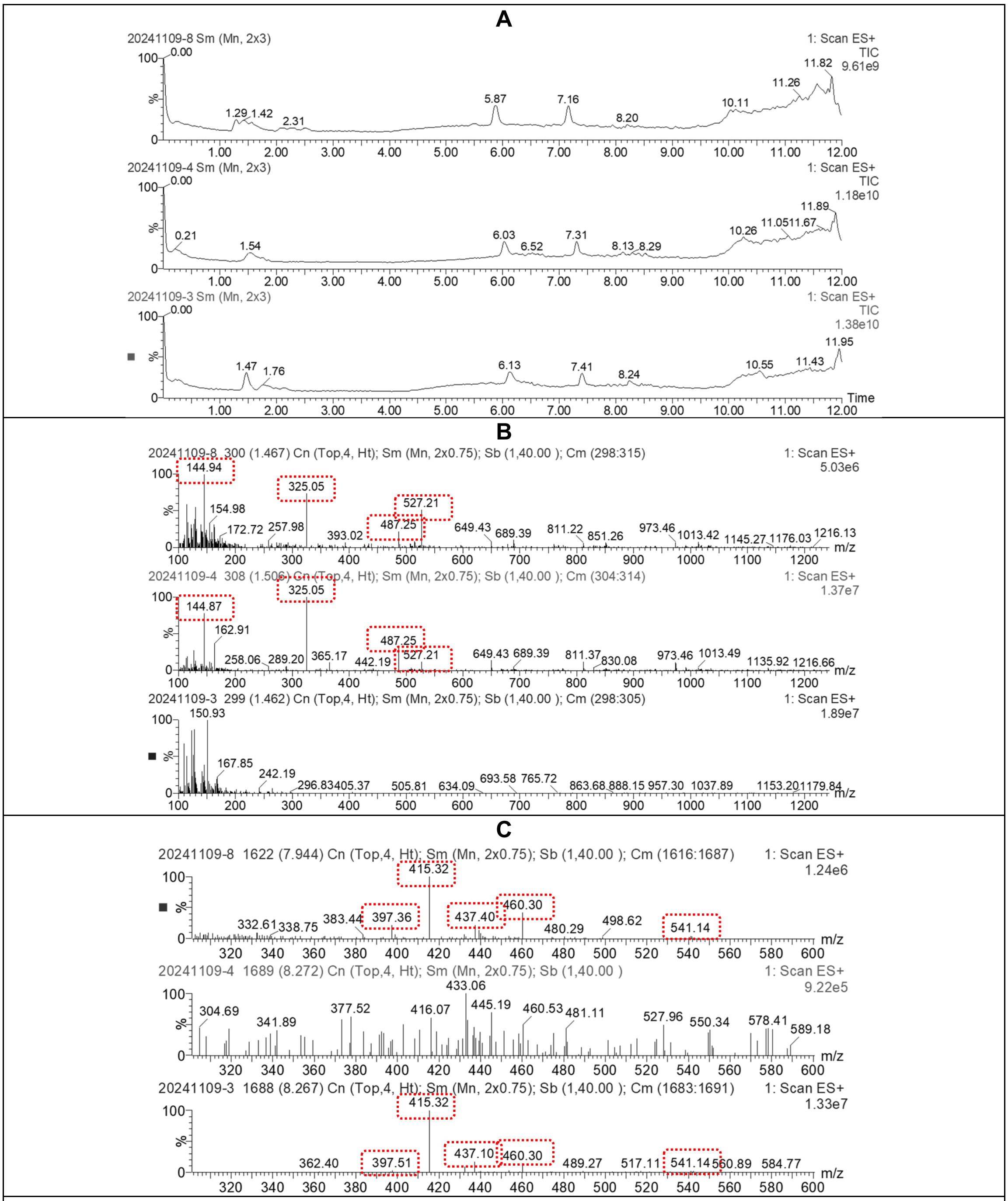

Figure S2. (A) LC-MS analysis (TIC-ESI+) of dietary supplement 10.0 (top), TheraPrime ingredient (middle) and FucoMax ingredient (bottom). **(B and C)** Extracted MS spectra of selected peaks at t_R 1.4 min and 8.1 min of each sample (in all cases, the MS spectrum of supplement 10.0 is at the top, TheraPrime at the middle, and FucoMax at the bottom). Red boxes highlight the same ions present in both samples.

2.1

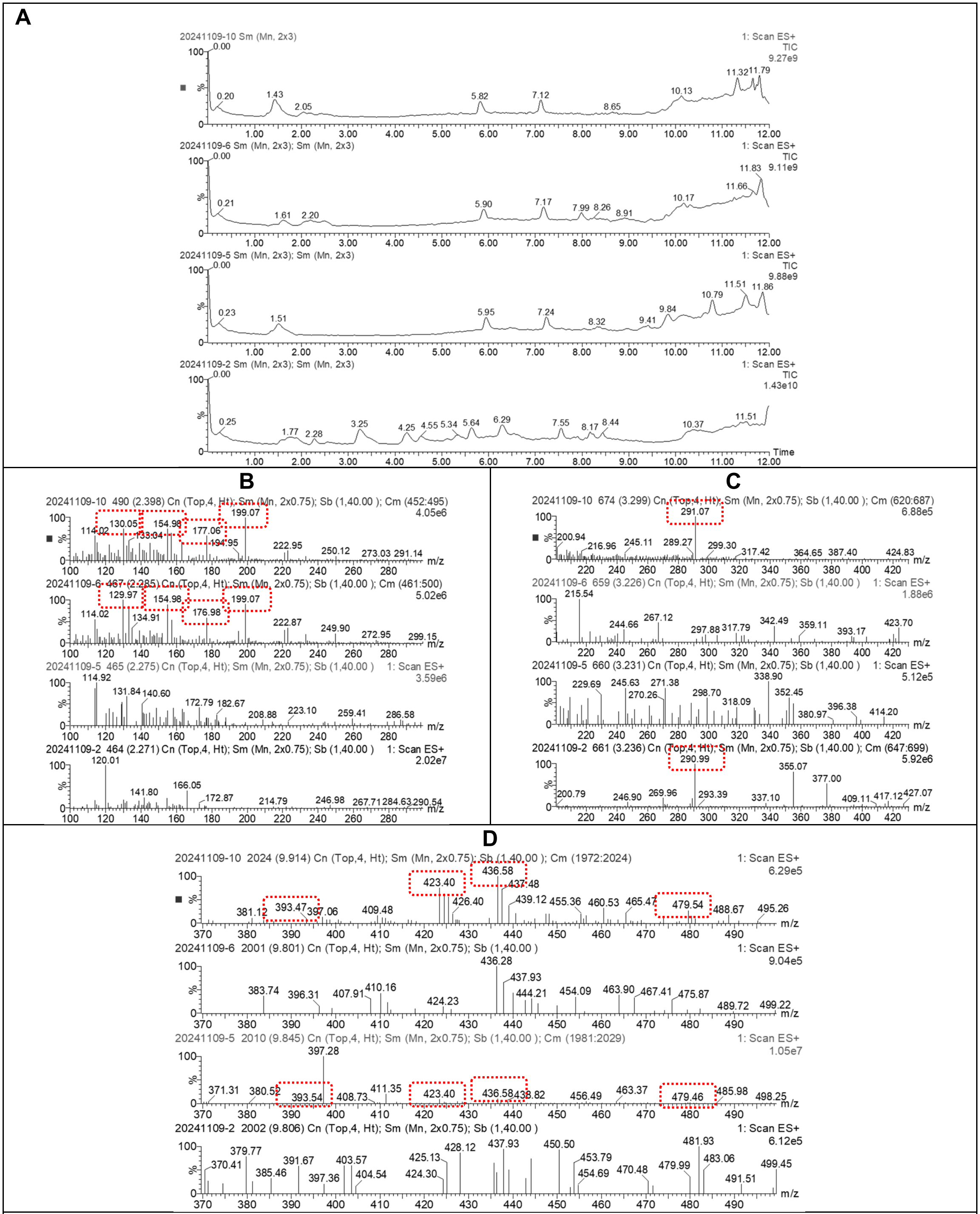

Figure S3. (A) LC-MS analysis (TIC-ESI+) of dietary supplement 2.1 and ThinOgen, AztaZine and ApplePhenon ingredients (from top to bottom). **(B-D)** Extracted MS spectra of selected peaks at tR 2.3 min, 3.2 min, and 9.8 min of each sample (in all cases, the MS spectrum of supplement 2.1 and ThinOgen, AztaZine and ApplePhenon are shown from top to bottom). Red boxes highlight the same ions present in both samples.

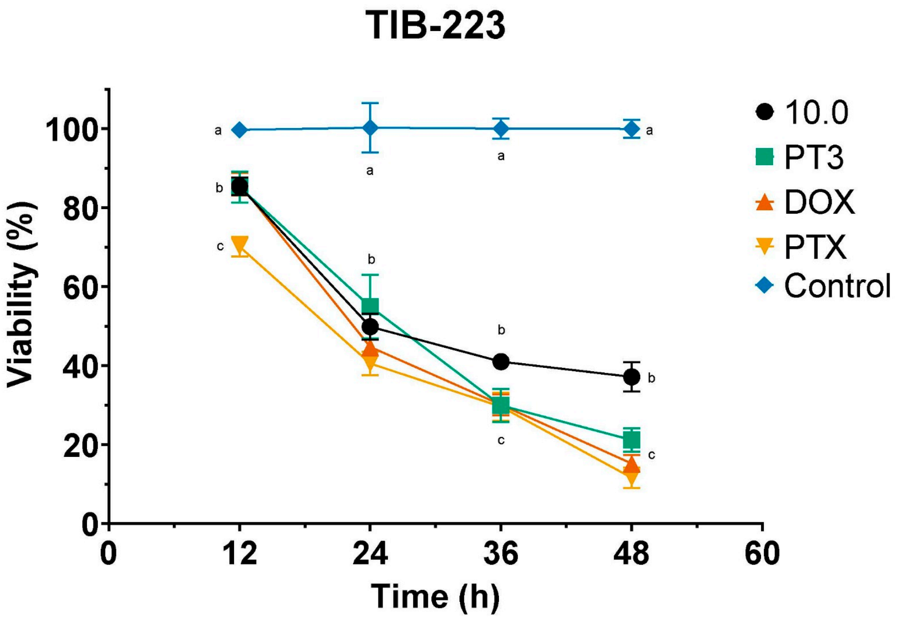

Figure S4. Time-dependent effects of PT3 and Supplement 10.0 on the viability of TIB-223 cancer cells. Cells were treated with each compound at their IC50 concentrations, and viability was assessed using the MTT assay at 12, 24, 36, and 48 hours. Untreated cells (blue line) served as the absolute control, while doxorubicin (DOX), and paclitaxel (PTX) were included as positive controls. Data represent the mean ± standard error (SE) of three independent experiments conducted in triplicate. Different letters (a-c) indicate statistically significant differences between treatments at the same time point, according to ANOVA followed by Tukey's test. A significance level (p-value) < 0.05 was considered statistically significant.

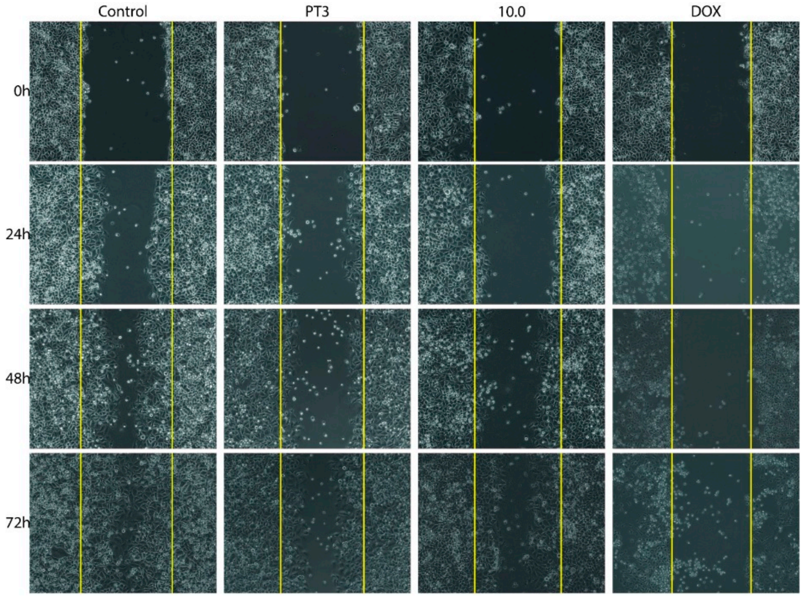

Figure S5. Wound healing assay using Caco2 cells. Images depict the monolayer of cancer cells post-wounding following treatment with plant tocotrienols (PT3), supplement 10.0, and doxorubicin (DOX; positive control) at their respective IC_{50} values, after 24, 48 and 72 hours. Untreated cells served as a negative control. The results presented are representative of three independent experiments.

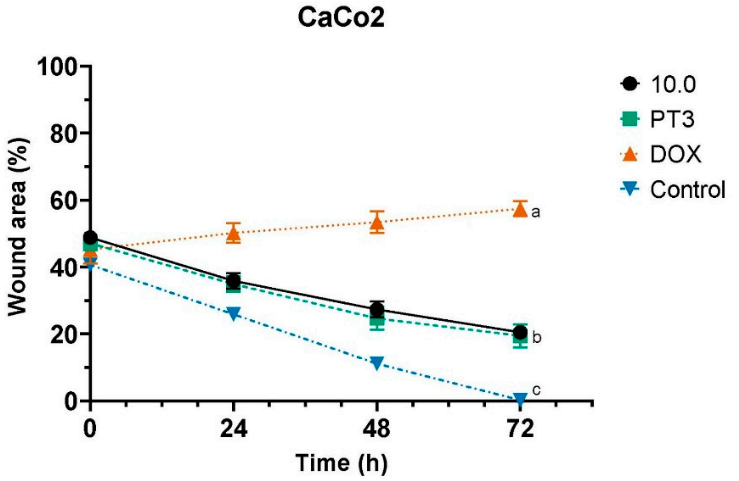

Figure S6. Change in wound area in the "Wound and Healing" assay of colon cancer cells. Caco2 cells were treated for 24, 48 and 72 hours with plant tocotrienols (PT3), Supplement 10.0 and doxorubicin (DOX). Untreated cells served as a negative control. Results are presented as the mean ± standard error of three independent experiments. Different letters (a-c) at 72 h indicate statistically significant differences according to ANOVA followed by Tukey's test. A significance level (p-value) < 0.05 was considered statistically significant.

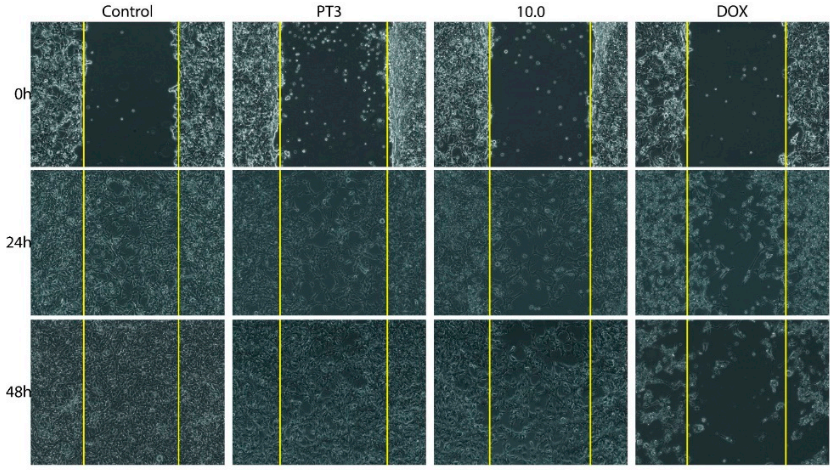

Figure S7. Wound healing assay using Detroit 548 cells. Images depict the monolayer of cancer cells post-wounding following treatment with plant tocotrienols (PT3), supplement 10.0, and doxorubicin (DOX; positive control) at their respective IC_{50} values, after 24, and 48 hours. Untreated cells served as a negative control. The results presented are representative of three independent experiments.

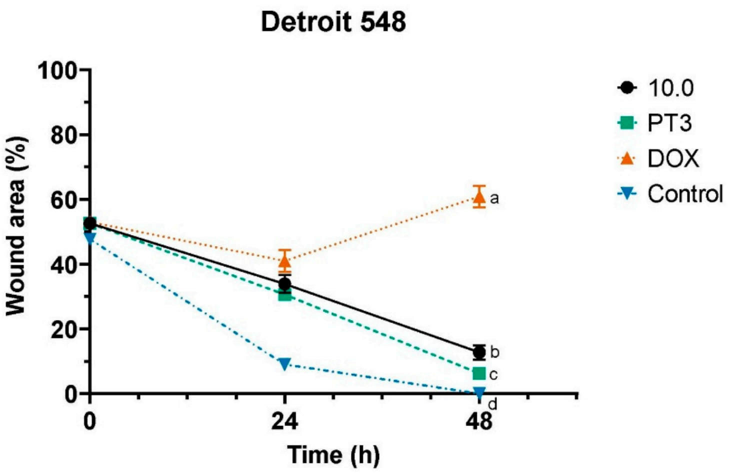

Figure S8. Change in wound area in the "Wound and Healing" assay of normal fibroblast cells. Detroit 548 cells were treated for 24 and 48 hours with plant tocotrienols (PT3), Supplement 10.0 and doxorubicin (DOX). Untreated cells served as a negative control. Results are presented as the mean ± standard error of three independent experiments. Different letters (a-d) at 48 h indicate significant differences determined by one-way ANOVA, followed by Tukey's post-hoc test. A significance level (p-value) < 0.05 was considered statistically significant.

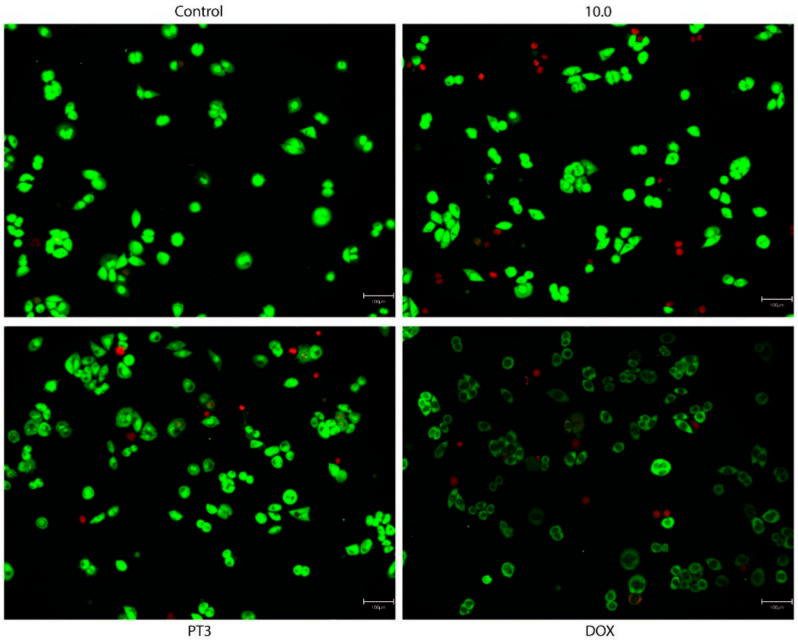

Figure S9. Double staining with AO/EB in Caco2 cancer cells. 10X magnification. Cells were stained with acridine orange and ethidium bromide (AO/EB) after 4 h of treatment at the corresponding IC_{50} concentration. Untreated cells served as a negative control. Cells treated with plant tocotrienols (PT3) and Supplement 10.0 showed apoptotic cells with condensed nucleus in yellow-green fluorescence by AO staining. (DOX; positive control).

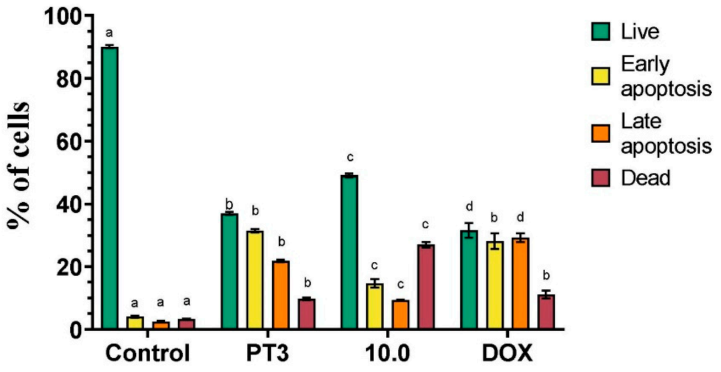

Figure S10. Induction of apoptosis in Caco2 cells treated with PT3, Supplement 10.0 and doxorubicin (DOX). The graph shows the percentage of live cells (green), early apoptotic cells (yellow), late apoptotic cells (orange), and dead cells (red) under different treatments with their corresponding IC_{50} values. Error bars represent the mean ± SEM from three separate experiments. Bars that have the same color, but showing different letters (a-d), indicate significant differences determined by one-way ANOVA followed by Tukey's post-hoc test. A significance level (p-value) < 0.05 was considered statistically significant.

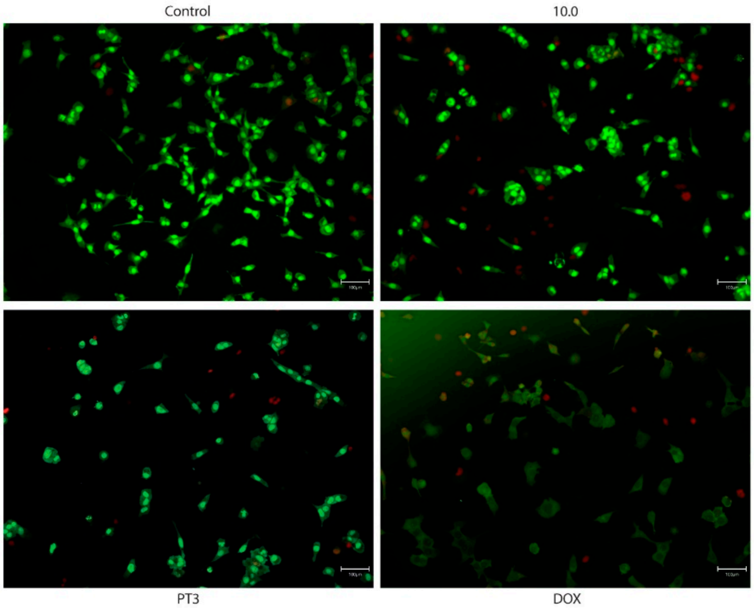

Figure S11. Double staining with AO/EB in Detroit 548 cells. 10X magnification. Cells were stained with acridine orange and ethidium bromide (AO/EB) after 4 h of treatment at the corresponding IC_{50}

concentration. Untreated cells served as a negative control. Cells treated with plant tocotrienols (PT3) and Supplement 10.0 showed apoptotic cells with condensed nucleus in yellow-green fluorescence by AO staining. (DOX; positive control).

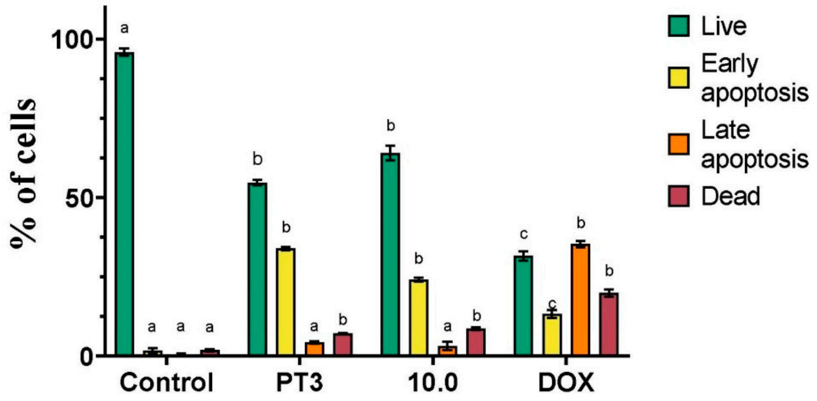

Figure S12. Induction of apoptosis in Detroit 548 cells treated with PT3, Supplement 10.0 and doxorubicin (DOX). The graph shows the percentage of live cells (green), early apoptotic cells (yellow), late apoptotic cells (orange), and dead cells (red) under different treatments with their corresponding IC_{50} values. Error bars represent the mean ± SEM from three separate experiments. Bars that have the same color, but showing different letters (a-d), indicate significant differences determined by one-way ANOVA followed by Tukey's post-hoc test. A significance level (p-value) < 0.05 was considered statistically significant.

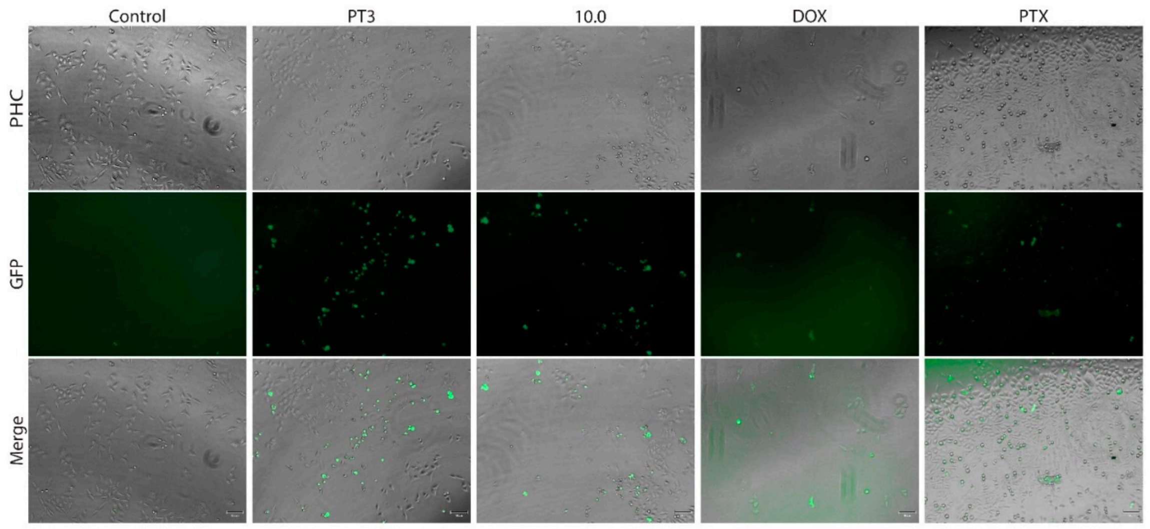

Figure S13. Caspase activity induced by PT3 and supplement 10.0 in colorectal cancer cells (Caco2). The activity of caspases 3 and 7 was determined by fluorescence microscopy. Phase contrast (PHC), GFP filter (GFP) and overlapping images (Merge) are shown. Doxorubicin (DOX) and paclitaxel (PTX) were evaluated as positive controls. Untreated cells served as the negative control. Each image represents a typical outcome from three separate experiments.

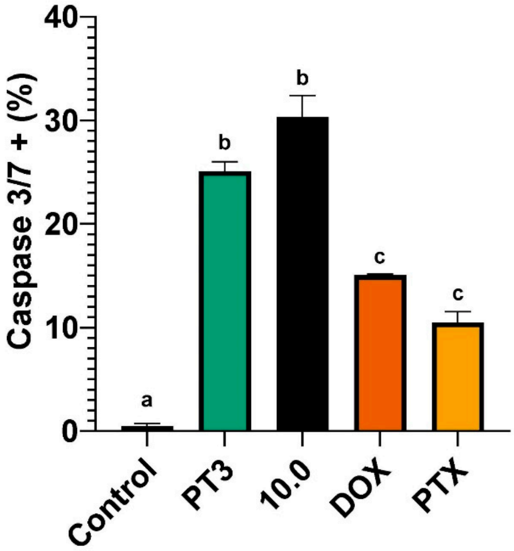

Figure S14. Caspase-3/7 activity in Caco2 cells treated with PT3, Supplement 10.0, doxorubicin and paclitaxel. The graph shows the percentage of caspase-3/7 positive cells in each treatment group. Error bars represent mean ± SEM from three independent experiments. Different letters (a-c) between treatments indicate significant differences determined by one-way ANOVA followed by Tukey's post-hoc test. A significance level (p-value) < 0.05 was considered statistically significant.

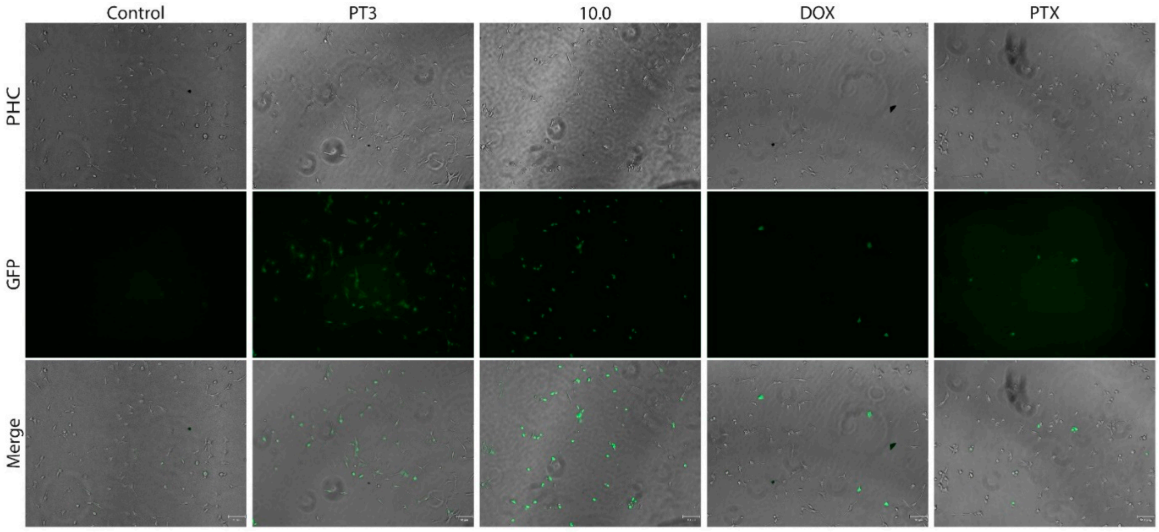

Figure S15. Caspase activity induced by PT3 and supplement 10.0 in fibroblast cells (Detroit 548). The activity of caspases 3 and 7 was determined by fluorescence microscopy. Phase contrast (PHC), GFP filter (GFP) and overlapping images (Merge) are shown. Doxorubicin (DOX) and paclitaxel (PTX) were evaluated as positive controls. Untreated cells served as the negative control. Each image represents a typical outcome from three separate experiments.

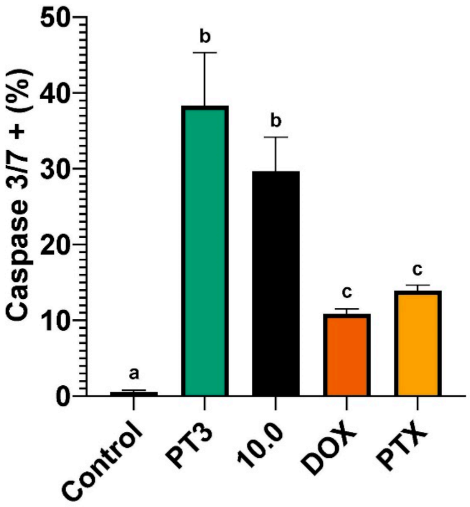

Figure S16. Caspase-3/7 activity in Detroit 548 cells treated with PT3, Supplement 10.0, doxorubicin and paclitaxel. The graph shows the percentage of caspase-3/7 positive cells in each treatment group. Error bars represent mean ± SEM from three independent experiments. Different letters (a-c) between treatments indicate significant differences determined by one-way ANOVA followed by Tukey's post-hoc test. A significance level (p-value) < 0.05 was considered statistically significant.
